# The Role and Impact of the Use of Information Technologies by Police in Response to Violence against Women

**DOI:** 10.3390/ijerph20126125

**Published:** 2023-06-14

**Authors:** Kimberly Przeszlowski, Rob T. Guerette, Lori K. Sudderth

**Affiliations:** 1 Department of Justice and Law, Quinnipiac University, Hamden, CT 06518, USA; lori.sudderth@quinnipiac.edu; 2 Department of Criminology and Criminal Justice, Florida International University, Miami, FL 33199, USA; guerette@fiu.edu

**Keywords:** violence against women, police technology, investigations, real-time crime centers, case clearances, sexual assault, domestic violence

## Abstract

The last decade has witnessed an increased awareness of the crucial need to enhance police response and investigation of crimes related to violence against women (VAW). Although some research has been conducted on police decision-making in response to these crimes, there remains a dearth of knowledge concerning the influence of innovative police technologies on the investigative process and resulting case outcomes. This knowledge gap is particularly concerning given (1) the intricate nature and severity of VAW crimes and (2) the substantial advancements in technology that have transformed how the criminal justice system handles violent crime cases. To address this gap, the current study adopted a multi-method, quasi-experimental design to assess the impact of the Miami Police Department’s Real-Time Crime Center (MRTCC) technologies on the case processing and case clearance of sexual assault and domestic violence incidents. The results of this study illuminate the distinctive features associated with this form of violent crime and underscore the necessity of continuously advancing the strategies employed to address these incidents.

## 1. Introduction

Sexual assault and domestic violence are two of the most serious and pervasive forms of violent crimes. Despite numerous efforts by law enforcement agencies and other stakeholders to combat these crimes, they remain a significant challenge for criminal justice systems across the country. One of the most significant issues in addressing these forms of violence against women (VAW) is the low clearance rates, which refers to the percentage of cases in which an arrest is made, or charges are filed. Improving case clearance rates is critical for both ensuring justice for victims and deterring offenders from committing these crimes. In recent years, clearance rates for these crimes have been lower than for other types of violent offenses. According to the FBI’s Uniform Crime Report, the clearance rate for rape, which includes sexual assault, was just 30 percent in 2020, compared to a clearance rate of 50 percent for murder and 69 percent for non-negligent manslaughter. The clearance rate for aggravated assault, which includes domestic violence, was 47 percent in 2020 [1].

The extant literature on this topic has suggested many reasons why case clearance rates for sexual assault and domestic violence are so low. Some of these noted impediments relate to cultural norms and stigma, while others relate to the limitations of investigative practice. For instance, cultural norms and societal attitudes towards women can contribute to low case clearance rates since, in some belief systems, violence against women is accepted or even condoned, making it more difficult for victims to come forward and for law enforcement to take action [2]. This phenomenon is also closely associated with the underreporting of incidents to law enforcement due to fears of retaliation or societal stigma, thereby further hindering our comprehensive understanding of the true magnitude of the problem [3].

Other reasons why case clearances for VAW are low relate to various aspects of police investigative practices. First, many cases fail to move forward due to a lack of evidence. Often, there is a lack of physical evidence that a violent encounter occurred, or there are no witnesses to the incident, which makes it difficult for law enforcement to proceed with an arrest or to bring charges for prosecution [4]. Second, some have recognized that police officers may not be adequately trained in how to properly investigate cases of violence against women. This can result in the mishandling of evidence, victim blaming, and failure to gather crucial information that could lead to successful prosecution [5,6]. Additionally, many police agencies operate with limited resources, which forces them to prioritize crime cases based on severity or likelihood of conviction. Given the challenges of victim cooperation and the absence of physical evidence or witnesses, these cases may be de-prioritized and receive less attention than others [7,8].

Taken together, this research suggests that there are a variety of factors that may serve to impede the successful arrest and prosecution of cases involving violence against women. Efforts to overcome these impediments may also work to improve the clearance of VAW cases. While many of these factors may be unique to this type of violence, an unmistakable need relates to the improvement of police investigative practice and methods to better identify, collect, and preserve evidence that can facilitate successful criminal justice processing. Improved collection and documentation of evidence could assist police and prosecutors in clearing cases where victim credibility may be limited, when there are no witnesses, or when the victim may be unwilling to cooperate.

One approach that has been increasingly adopted by local police agencies in recent years to improve the collection of evidence and assist in the investigation of crimes is the development and use of centralized technological units housed within so-called “real-time crime centers” [9]. RTCCs are dedicated facilities that use advanced technologies to collect, analyze, and disseminate data and information about criminal activity to responding police and investigators prior to, during, and immediately following crime incidents. These centers typically employ a multitude of information technologies, which include CCTV cameras, gunshot and acoustic sensors in violent crime areas, vehicle license plate readers, and a host of informational databases and automated search software. These technologies are used together by RTCC detectives and allow for the documentation of evidence of offenders either committing the crime, entering, or exiting the crime scene, or providing other circumstantial evidence, leading to a suspect’s identification and subsequent collection of evidence obtained through warrant searches.

Relatively new, emerging research has found that the use of RTCC technologies has improved the ability of police to solve violent crimes [10] and reduced crime levels, particularly sexual assaults, within specific communities [11]. However, the one study that examined their impact on case clearances only looked at the effect of RTCC technologies on all violent crime types. It remains unclear whether the use of these technologies can improve clearances of incidents involving violence against women. This study seeks to examine this topic further with a quasi-experimental study that examined the influence of RTCC technologies on case clearances of domestic violence and sexual assaults in relation to other violent crime types. The study also assesses the role of RTCC technologies within the investigation of incidents to better understand their potential influence in VAW case processing.

### 1.1. Research Context

Two pertinent areas of research are currently being explored in the realm of improving the response and outcomes of incidents of violence against women, both of which focus on the impact of police practices. The first area of investigation concerns the traditional or extralegal factors that are involved in the processing of cases, while the second area of study examines the influence of police technology on the outcomes of such investigations. A comprehensive review of each of these areas is necessary to provide a nuanced understanding of the complex factors that contribute to the effectiveness of policing interventions in this domain. Following this review, a brief overview of the Miami Real-Time Crime Center is presented, which serves to contextualize and illustrate the practical implications of the research findings.

### 1.2. Factors That Contribute to VAW Case Clearance

When survivors do report to the police, they are often motivated by a desire to prevent the perpetrator from victimizing others or because they have received encouragement and support from formal or informal sources to report the crime to law enforcement authorities [12]. However, despite the high incidence of crimes that constitute VAW, such as sexual assault and domestic violence, a substantial proportion of cases (estimated at 32–35%) are not reported to the police [3]. This underreporting phenomenon has prompted scholars to examine the role of trust in law enforcement authorities, who are often the primary point of contact for victims, in shaping their decision to report sexual assault [13,14].

The decision to arrest in sexual assault cases has been linked to the suspect’s history of assaulting the victim, their relationship with the victim, injuries sustained by the victim as a result of the assault, as well as the resistance reported by the victim, weapon use, the presence of evidence, including witnesses, a forensic exam, and suspect interview, as well as how quickly the victim reported the rape or abuse to the police [4,15]. Thus, paramount in the arrest and prosecution of these cases is the continued cooperation of the victim [4,15,16,17]. Extralegal factors, especially those related to the credibility of the victim, have been related to the likelihood of arrest in some studies but not others (see Lapsey, Campbell, and Plumlee, 2022 for a review of the literature).

Given that victim engagement is critical to the successful prosecution of these cases, some researchers have investigated the factors that encourage or discourage victims to continue with the criminal justice process. Victim continuation, for example, may be contingent upon the victim or situational characteristics—mental health, how confident they feel about the case, desire to stop the offender or relationship with the offender, presence, or absence of social support [12,18]. Victim continuation may also be dependent on criminal justice system characteristics—the quality of interactions with police and other criminal justice personnel, police culture that communicates suspicion, and para-military expectations [17,19,20,21]. Rumney and McPhee (2023), for example, found in a British study that when police provided practical assistance, time and space for victims to make decisions, and allowed them to feel in control of the process, victims were more satisfied, and often continued to engage with the case, even if it meant coming back later to pursue the successful prosecution of the offender [17].

### 1.3. Impact of Police Technology

Violent incidents against women pose unique challenges to criminal investigations due to their sensitive and personal nature. Unlike other forms of criminal offenses, these crimes involve a complex interplay of psychological and emotional factors, which can complicate the investigation process. Moreover, the physical evidence collected is often scarce, making it more challenging to establish guilt beyond a reasonable doubt. Even in cases where such evidence is available, and despite the tremendous utility of forensics (e.g., sexual assault kits), evidence too frequently goes unused or becomes backlogged within the system [22,23]. Consequently, investigators have traditionally heavily relied on victim testimony and other forms of circumstantial evidence to build a case.

In recent years, police investigations have undergone significant advancements, leading to a noteworthy impact on the ability to solve crimes. Largely facilitated by the reauthorization of the Violence Against Woman Act (VAWA), several additional measures have been implemented to enhance the effectiveness of investigations, including the establishment of specialized police units, sensitivity training for law enforcement personnel, and the formation of sexual assault response teams, among other initiatives [24,25,26]. The progress of technology has proven to be crucial in augmenting police investigations of these incidents. One such innovation, the use of body-worn cameras (BWCs), has emerged as a promising tool for enhancing the evidence-collection process. BWCs offer the capability to record a victim’s emotionally charged statement, document the physical context of an incident, and preserve witness testimony, all of which can significantly bolster the quality of evidence available to law enforcement agencies [27].

The evidentiary significance of digital technologies has also become widely recognized. The conventional explanation for the low rate of arrests and convictions in cases of sexual assault or domestic violence is the difficulty in obtaining conclusive evidence due to conflicting testimonies [28,29,30]. The emergence of digital evidence, however, challenges this argument. Digital evidence includes social media exchanges between the victim and perpetrator, as well as mobile phone videos and photos that offer critical contextual information about the crime [31]. The shift towards gathering digital evidence, previously limited to external private CCTV, has now become a crucial investigative tool that is comparable to more traditional forms of forensic evidence, such as DNA and fingerprints [31,32].

While it is commonly accepted that closed-circuit television (CCTV) systems can aid investigators in addressing key inquiries that arise during criminal investigations, the impact of such technologies on the investigative outcomes of VAW incidents has received limited scholarly attention. This lack of research is not unexpected, considering that these crimes often occur in residential settings outside the purview of CCTV cameras. Studies that do manage to include this category of crime in their analyses suggest that CCTV technology has the potential to serve as a valuable tool in the investigative process. For instance, Ashby (2017) discovered that CCTV was significantly associated with an increased likelihood of crime resolution in all categories of crimes except drug or weapon possessions and fraud [33]. Notably, for sexual assault offenses, CCTV remained linked with significantly higher availability and detection rates and provided valuable evidentiary information in 18% of all cases.

Of particular interest to this research is the role and impact of rapidly diffusing RTCC information technologies on these crimes. RTCCs are investigative units that exhibit varying structures but are generally characterized as data platforms that promptly synthesize and provide officers with comprehensive intelligence information. Staffed individuals within RTCCs possess the ability to actively monitor critical infrastructure and assets, generate post-incident investigative evidence, and facilitate cooperation across both internal units and external agencies. The quality of the information provided to officers, detectives, and external partners, as well as the turnaround time from incident to evidence, has established these units as highly valued resources for crime control and investigative purposes. At present, the evaluations of RTCCs are limited to the Chicago Police Department’s Strategic Decision Support Centers (SDSCs) and the Miami Police Department’s centralized Real-Time Crime Center (MRTCC). While the former evaluation discovered some evidence of success in decreasing sexual assaults in districts that implemented SDSCs [11], the latter did not assess VAW in isolation, but rather found support for enhanced crime clearance rates for all forms of violent crime [10].

## 2. Materials and Methods

### 2.1. Miami Real-Time Crime Center Overview

The MRTCC was established in April 2019 as a fundamental component of the Criminal Investigations Division’s Investigative Support Section. The main objective was to provide real-time, actionable investigative intelligence, enhance situational awareness, and generate a virtual environment for law enforcement personnel responding to priority calls and unfolding events. To accomplish this goal, the MRTCC was designed to manage and coordinate the operations of various technological systems, such as CCTV cameras, gunshot detection software, license plate readers, facial recognition, and social media software. Over the course of the study period, the MRTCC gradually expanded its capabilities to operate approximately 582 cameras and access a wide range of information technology resources. The unit was staffed with nine full-time personnel, providing coverage for 86% of weekly hours, equivalent to near 24/7 operations.

### 2.2. City Characteristics

Miami is an urban area with a high population density, covering approximately 36 square miles and inhabited by a diverse population of roughly 450,000 people. The city’s demographic makeup comprises 70% Hispanic, 16% African American/Black, and 14% White Non-Hispanic individuals. Over time, Miami has undergone significant growth as a metropolitan hub and has become a popular tourist destination with a tourist-to-local ratio of nearly 1600 visitors per 100 residents. However, despite its growth and prosperity, poverty levels in the area remain higher than the national average. Like many other large cities, crime tends to be concentrated in specific neighborhoods, which has led to the predominant deployment of MRTCC technologies in these areas.

### 2.3. Research Focus

The primary objective of this study was to examine the impact of police information technologies on the outcomes of cases that involve VAW incidents and to assess their role within the investigative process. Specifically, the study aimed to assess whether the use of MRTCC technologies was associated with higher rates of case clearance and to what extent these technologies were integrated into the processing of sexual assault and domestic violence cases, as compared to other forms of violent crimes and traditional evidence sources. The research questions guiding this investigation were twofold: (1) what is the influence of MRTCC technologies on the clearance of VAW incidents? and (2) to what extent have MRTCC technologies been integrated into the case processing of VAW incidents?

The decreasing incidence of sexual assault and domestic violence incidents in “offline” settings, coupled with the increasing importance of digital evidence in linking victims and alleged perpetrators, suggest that the police agencies’ technological resources could enhance the investigation of these crimes [28]. This hypothesis is also supported by prior research, which indicates that using a wide range of sources of evidence in response to violent crime incidents improves the ability to identify offenders and achieve higher case clearance rates [10].

### 2.4. Analytical Approach

In the current study, three different analytical techniques were utilized to determine the extent to which RTCC technologies impact the investigation of violent crimes against women and their outcomes. First, a set of nested multivariate logistic regression models were employed to determine the likelihood of case clearance for sexual assaults and domestic violence incidents compared to all violent crime incidents and controlling for RTCC support. The multivariate model was adjusted for various case characteristics and solvability factors that could potentially impact the clearance probability of incidents outside the scope of the technologies employed within the unit.

Second, module network analysis was used to empirically examine the extent to which RTCC technologies influence the investigative process of these unique crime types. This type of network analysis involves a set of integrated techniques that aim to depict the relational properties of individual elements and analyze the structures that emerge from the recurrence of these relations [34]. The network modules employed in this study were constructed using an EBICglasso estimation procedure coupled with a non-paranormal transformation. EBICglasso represents a robust statistical methodology for conducting network analysis on datasets characterized by high dimensionality. Its primary strength lies in accurately estimating the conditional dependence relationships between variables, a critical aspect in network analysis [35]. One notable advantage of this approach is its efficient determination of the sparsity pattern of the graphical model, enabling the identification of significant connections while discarding insignificant ones. Furthermore, EBICglasso demonstrates proficiency in handling scenarios where the number of variables significantly surpasses the number of observations, a common occurrence in fields reliant on high-throughput data [36].

Finally, a post hoc analysis utilizing non-nested logistic regression was undertaken to discern the impact of conventional extralegal predictors on case clearance rates for instances of sexual assault and domestic violence. These extralegal factors encompass the relationship between the victim and the suspect, the incident’s location, and the severity of the crime. It is worth noting that not all instances of violent crime provided data pertaining to these factors. For a comprehensive understanding of the data collection methodology, please consult the “Post Hoc Analysis” section outlined below.

### 2.5. Data

The dataset utilized for this study stemmed from a collaborative Smart Policing Initiative with the City of Miami Police Department, which concluded in October of 2022. (This research was supported by grant number 2019-WY-BX-0005 awarded by the USDOJ, Bureau of Justice Assistance. Points of view or opinions in this document are those of the authors and do not necessarily represent the official position or policies of the U.S. Department of Justice). The analyses relied on data from an expanded MRTCC incident log analytical database, which contained measures of violent crime incident characteristics and sources of evidence used by responding officers and detectives within the unit. An initial MRTCC incident log established within the unit recorded basic information on every incident to which the unit assisted. This section will detail the process involved in gathering this information, with emphasis on the measures included for analyses that follow.

### 2.6. Internal MRTCC Log

The MPD RTCC maintained an internal log to document their daily activities and work associated with requested incident support. The incidents recorded within the log provided a framework for the development of the extended log used in the eventual outcome evaluation of the unit. The log-recorded incidents brought to the attention of MRTCC personnel through dispatch or officers in the field incidents identified through active monitoring, and cases in which the MRTCC was asked to assist in the post-incident investigative process. The assembly of the internal log began in January 2020 and at the time of the evaluation period, it consisted of 3636 entries, of which 2228 involved an incident resulting in an assigned case number.

### 2.7. Expanded Analytical Data Log (Treatment Group)

The purpose of the expanded database was to capture additional measures for all violent and property crime incidents that received support from the MRTCC. The incidents recorded within the expanded MRTCC database had all received real-time or post-hoc deployment of information from the MRTCC to responding officers or detectives. The extended database allowed for more in-depth analysis of the assessment of selected outcomes for incidents treated by the RTCC compared to non-MRTCC incidents. The data collection for the extended MRTCC analytical database occurred simultaneously with the internal MRTCC incident log. Data collection was concluded on 30 April 2022, to allow for the assembly of the non-MRTCC treated control sample. The violent crime incidents recorded within the extended MRTCC database are comprehensive. That is, all homicides, aggravated assaults, robberies, sexual assaults, and domestic violence incidents that were included and validated within the internal MRTCC log were also captured within the extended database. Selected property crimes (e.g., burglaries, larceny thefts, vandalism) were likewise included within the extended database. The extended analytical database consisted of a total number of 886 incidents, of which 648 were violent crimes. The time period of cases within the expanded MRTCC database was naturally the same as that in the MRTCC incident log.

The selected measures included victim characteristics (victim age, sex, and race), human-based evidence sources (the presence of a victim interview, witness interview, lineup, public flyer, or the presence of a civilian-owned CCTV system), crime scene evidence (whether a gun or vehicle was used, gunshot residue was searched for, Latent prints or DNA were searched for, the crime scene investigation (CSI) team was deployed, and whether ballistics tracing via NIBIN was undertaken), and warrant evidence and manpower used (whether a search warrant was issued, a cell warrant was issued, and the number of officers who worked on the case).

All but two of these measures were coded dichotomously with 1, indicating it was present within the investigation of the case, and 0 indicating its absence. Two measures, victim age and the number of officers responding, were coded as continuous measures. There were two dependent measures in the outcome analysis, whether the case was cleared by arrest (coded as 1 indicating an arrest was made and 0 one was not) and the number of days from the time the incident occurred to the time the arrest was made. Days to arrest was coded as a continuous measure. Each of these variables had precedent in the literature as representing factors that were relevant predictors of crime solvability [37,38,39].

### 2.8. Control Sample

A randomly drawn control sample was also used to compare differences in these outcomes across the two samples while adjusting for violent crime incident characteristics. The control sample was stratified by neighborhood operational area and violent crime type proportionate to that represented in the MRTCC “treated” expanded analytical database. This random selection within the stratifications resulted in equivalency between the two samples based on those two stratifications. There were no significant differences between the two in terms of the number of incidents occurring in the respective neighborhood operational areas or the number of violent crime incident types. Having proportionate numbers of incidents occurring within specific neighborhood areas and incident types ensured that neighborhood effects (usually measured by neighborhood demographics) were equivalent between the two samples. The control sample included the same dependent measures and incident characteristic measures that were recorded in the MRTCC expanded analytical database and were coded in the exact same manner.

## 3. Results

Table 1 presents an overview comparison of the measures utilized in the nested multivariate logistic regression analysis and module network analysis. This comparison includes both the MRTCC-assisted cases and control cases for the complete violent crime model, which encompasses a range of violent crime types, including homicides (n = 120), aggravated assault (n = 543), robberies (n = 439), sexual assaults (n = 42), and domestic violence incidents (n = 84). Furthermore, the table highlights any discrepancies in measures specifically related to sexual assaults and domestic violence incidents when they are analyzed in an isolated manner.

### 3.1. Full Violent Crime Model

For the outcome measure, a larger proportion of cases from the MRTCC-assisted sample (N = 237, 39%) were cleared compared to the control sample, where only 202 cases (33%) were cleared. Among human evidence characteristics, four out of five measures were significantly different between the two samples. The MRTCC-assisted cases had a statistically significant greater proportion of witness interviews (n = 221 vs. n = 190), conducted more suspect lineups (n = 141 vs. n = 102), and disseminated more public information flyers (n = 191 vs. n = 54). The presence of external or private CCTV footage was also significantly higher in the MRTCC-assisted cases (n = 286 vs. n = 71). Concerning crime scene-based evidence, five out of six measures were significantly different between the samples. The MRTCC-assisted cases had significantly more guns present (n = 292 vs. n = 185) and vehicles used (n = 353 vs. n = 189) in the commission of a crime, leading to more processing of gunshot residue (n = 72 vs. n = 22), greater use of the CSI unit (n = 327 vs. n = 228), and more utilization of NIBIN ballistics tracing (n = 171 vs. n = 42). Finally, regarding warrant evidence and manpower, there were significantly greater numbers of search warrants (n = 47 vs. n = 12) and cell warrants (n = 38 vs. n = 9) used, as well as more officers assigned to incidents (mean = 4.02 vs. mean = 3.74) in the MRTCC-assisted cases than in the control sample.

### 3.2. Violent Crimes against Women

Once observed independently, there were no significant difference in the case clearance outcome measure between the MRTCC-assisted cases and the control sample for both sexual assault incidents (n = 6 vs. n = 4, respectively) and domestic violence incidents (n = 36 vs. n = 33, respectively). For sexual assault incidents, only three evidence sources exhibited a significant difference between the MRTCC-assisted cases and control cases. These evidence sources comprised the use of a suspect lineup (n = 7 vs. n = 17), the presence of external CCTV evidence (n = 10 vs. n = 2), and whether a vehicle was used in the commission of a crime (n = 10 vs. n = 1). For domestic violence incidents, two evidence sources exhibited significant differences, that being the age of the victim, with the control sample being slightly older (mean = 37 vs. mean = 32), and whether a vehicle was used in the commission of the crime, with a higher frequency of vehicle usage depicted in the MRTCC-assisted cases (57% vs. 17%).

### 3.3. Case Clearance

Table 2 presents the analysis results aimed at examining whether MRTCC technologies impacted the case clearance of violent crimes against women. Although Table 1 statistics showed that MRTCC-assisted sexual assault and domestic violence incidents were cleared more frequently, these numbers alone do not consider the differences in the case processing of these crimes. Therefore, to accurately determine the impact of MRTCC technologies on clearance rates for these VAW incidents, the multivariate logistic regression analysis controlled for such differences. This analysis yielded noteworthy results, indicating that the presence of MRTCC technologies remained a significant predictor of case clearance for domestic violence incidents, even after accounting for variations in victim characteristics and the intricacies of case processing across subsequent models. However, it is important to note that the utilization of MRTCC technologies did not exhibit a significant impact on case clearance rates for sexual assaults. Instead, the analysis unveiled that the employment of MRTCC technologies significantly diminished the odds of case clearance for sexual assault incidents. Consequently, solely the final model will be presented herein.

In Model Six, all variables, including those related to warrant evidence and manpower, were included in the final analysis. The results showed that sexual assault incidents had an 80% lower probability of being cleared when compared to other violent crime types and case characteristics (*p* = ≤0.001). Conversely, domestic violence incidents were ten times more likely to be cleared (*p* = ≤0.001). Among the array of variables considered, the utilization of MRTCC technology, witness interviews, lineups, suspect flyers, and search warrants emerged as significant contributors to enhancing the odds of case clearance. Notably, the most influential predictor among them was the execution of a suspect lineup, which amplified the odds of case clearance by a factor of five. Conversely, the presence of external CCTV surveillance and the involvement of firearms exhibited a substantial decrease in the likelihood of case clearance, taking into account all other factors pertaining to solvability.

The chi-square and −2 log-likelihood coefficients suggested all six models were statistically significant, indicating a good model fit, and Nagelkerke pseudo-R-square indicated the increasing ability of the models to predict the model variance ranging from a low of nine percent in Model One to a high of twenty-nine percent in Model Six. All independent measures used in these models were examined for multicollinearity using the variance inflation factor (VIF) with no multicollinearity revealed. All VIF factors ranged from a low of 1.098 to a highest of 2.091 (these scores are far below the area of concern for VIF where convention dictates that scores of 5 or greater suggest the presence of multi-collinearity among independent measures).

### 3.4. Case Processing

Module network analysis helped to determine the influence of MRTCC technologies on the case processing of sexual assault and domestic violence incidents. The analyzed data were drawn from the expanded data set derived from the MRTCC log and the stratified randomly drawn control sample as discussed in the previous “Data” section, with a total sample size of 1222 included within the analysis. The data were coded to represent whether various evidential sources were used in the investigation of the violent crime incident, regardless of whether usable evidence was obtained from the evidence sources. These evidence sources were grouped into four types and included MRTCC evidence (CCTV, LPR, social media, facial recognition, and ShotSpotter), crime scene evidence (NIBIN, GSR, CSI, Latents/DNA), human-based evidence (victim interviews, witness interviews, media flyers, lineups, and external CCTV) and warrant-based evidence (search warrants, cell warrants (for an overview of the measures used in this analysis, refer back to the comparative overview of both samples presented in the previous table). Two additional crime type variables were intentionally coded to represent the investigation of VAW incidents with the aim of examining the role of police technology in the processing of these crimes. These nodes are maintained within the violent crimes against women category. Victim characteristics and other investigative solvability factors were not included as they were outside of the scope of the analysis.

**Figure 1:**
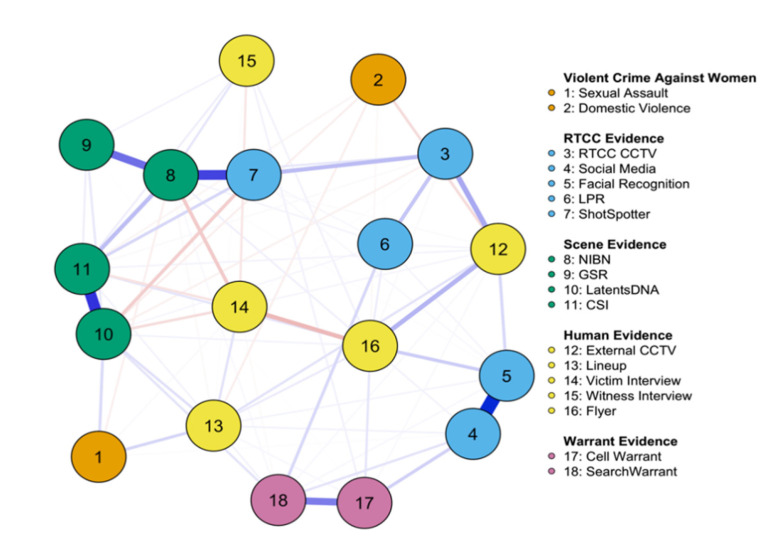
Module Network of MRTCC—Assisted Violent Crime and VAW Incidents.

Figure 1 displays an 18-node network with 79 out of 144 possible edges, resulting in a sparsity measure of 0.484. This measure quantifies the difference between a fully connected network, where all nodes have a relationship with one another, and the actual number of relationships observed in the analyzed network. A lower sparsity metric suggests greater interconnectedness among nodes, while a higher metric indicates weaker connectivity. In this case, the network is moderately dense, as supported by the number of non-zero edges present in the matrix. However, the role of MRTCC technologies in the investigation of these crimes is less prominent, and their use appears to be contingent on the existence of established connections within the network.

Notably, and as expected based on the existing body of literature, the sexual assault incident node is primarily linked to the presence of suspect lineups latent/DNA testing, and victim interviews, with weaker connections established with external CCTV and NIBIN. The sole relationship observed between MRTCC technologies, and the sexual assault node is linked to social media searches and identifications. It is plausible that these searches on social media platforms are conducted by MRTCC personnel to gather supplementary information regarding the individuals under investigation. For instance, such searches may aid in tracking suspect movements or identifying specific items of clothing worn during the commission of the crime. In contrast, investigations related to domestic violence cases heavily rely on nodes representing external or private CCTV systems, followed by suspect lineups, latent/DNA testing, and crime scene investigation processing. Of all the MRTCC technologies examined, the LPR system exhibited the greatest influence on the domestic violence node. This can be observed in Table 1, where it becomes apparent that a larger number of vehicles are involved in domestic violence incidents compared to sexual assault cases.

**Figure 2:**
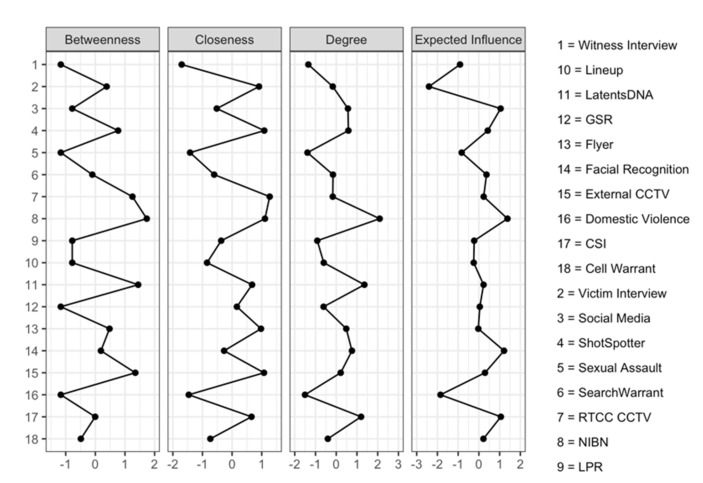
Centrality Plots of MRTCC—Assisted Violent Crime and VAW Incidents.

Furthermore, understanding the centrality of nodes within the network holds significance, as nodes with high centrality function as pivotal hubs connecting other nodes, while those with low centrality possess weaker peripheral connections. Four commonly employed indices, namely betweenness, closeness, degree, and expected influence, are employed to gauge node centrality. Collectively, these indices offer a quantitative assessment of a node’s significance within the network. As depicted in Figure 2, the centrality plots reinforce the limited integration of MRTCC technologies in the investigation of VAW incidents. Instead, we observe a greater endorsement for the utilization of MRTCC technologies in the investigation of other forms of violent crimes, with VAW incidents remaining situated on the periphery of the network.

### 3.5. Post Hoc Analysis

A post hoc analysis was conducted to determine the influence of traditional extralegal predictors of case clearance for both sexual assault (n = 42) and domestic violence incidents (n = 84). To perform this analysis, additional variables were collected at a later stage using the same methodology as outlined in the “Data” section. The information retrieved was obtained from the case file of each incident itself, in which the initial report was completed at the time of an officer’s arrival at the scene and updated by an investigator assigned to the sexual assault or domestic violence unit. Thus, the difference in data collection periods should not impact the results of the analysis, as these are extralegal variables that remain stable in the investigation over time.

These variables were grouped into the following four distinct categories: victim characteristics (victim, victim gender (female), victim race (Black), victim–offender relationship (family, intimate partner, or stranger), location (public space, residence), and crime severity (weapon used, visible injury, under the influence, prior record). These variables can be found in precedent literature as important factors impacting the case outcomes of VAW incidents [24,40,41]. All measures, aside from victim age, were coded dichotomously, with 1 indicating its presence in a case and 0 indicating its absence. The dependent measure in the analysis was whether the case was cleared by arrest (coded as 1 indicating an arrest was made and 0, one was not). As indicated in Table 3, there were no significant differences in any of the sexual assault variables between the MRTCC-assisted cases and the control cases. With respect to domestic violence, the age of the victims within the MRTCC-assisted cases (n = 32) was considered to be significantly lower than the age of victims within the control cases (n = 37), albeit the difference being five years. Furthermore, as evidenced within the table, both the MRTCC-assisted cases and the control cases in domestic violence incidents were perpetrated by an intimate partner or family member, rather than a stranger.

### 3.6. Extralegal Predictors of Case Clearance

Table 4 presents the results of the analysis examining whether extralegal factors influenced the odds of case clearance for sexual assault and domestic violence incidents. While the previous analyses suggested that police technology played a limited role in these crimes, it was unclear how other solvability factors, beyond victim characteristics, impacted case clearance. Therefore, a non-nested multivariate logistic regression analysis was conducted, adjusting for these additional variables to provide a more accurate depiction of their influence. Due to the small sample size (N = 126), a nested model was not appropriate to test the influence of all 14 independent variables.

Despite including the victim characteristics, victim–offender relationship, location, and crime severity variables, the analysis consistently showed significantly lower odds of case clearance for sexual assaults compared to domestic violence incidents. The likelihood of arrest for sexual assaults remained between 93% and 95% lower than for domestic violence incidents. Additionally, none of the covariates included in the analysis, beyond the sexual assault variable, reached statistical significance. Although all six models were statistically significant according to the chi-square and −2 log-likelihood coefficients, Model Six predicted the model variance best, with a Nagelkerke pseudo-R-square value of forty-three percent.

## 4. Discussion

The current study adopted a multi-method, quasi-experimental design to assess the impact of the Miami Police Department’s RTCC technologies on the case processing and case clearance of sexual assault and domestic violence incidents. The present study builds upon prior evaluations of RTCCs conducted in the districts of the Chicago Police Department [11], as well as the analysis of crime clearance rates in the City of Miami, which utilized a comprehensive model encompassing all types of violent crimes [10]. The findings of this study underscore the significance of closely examining diverse forms of crime in a more isolated manner. Depending on the distinctive attributes associated with each category of crime, specific technological approaches may prove more advantageous than others. For instance, in the case of crimes motivated by personal reasons or crimes of passion, the availability of solvability factors may inherently differ from those cases that do not cause harm to a victim or exhibit distinct motivations.

The study yielded three primary findings. First, despite the broad array of information technologies housed within the unit, their impact on the case clearance of sexual assault incidents is limited. Irrespective of the models assessed, sexual assault cases were less likely to be cleared than other forms of violent crime. This may be due to the complexity of investigating sexual assaults, which presents unique challenges for law enforcement, including the private nature of the crime, lack of eyewitnesses, and difficulties in obtaining physical evidence and credible testimonies. Without some form of digital evidence, the processing of a case through the real-time crime center seldom occurs. Inconsistencies in victim and witness statements and the difficulty in collecting and processing physical evidence, especially if the assault happened outside of a specific time frame, may also contribute to this outcome. Consequently, due to underlying factors that may further complicate a sexual assault case, the adoption of centralized police technologies alone may not have a significant impact on the case clearance rates of sexual assault incidents.

Secondly, the findings of the study indicate that domestic violence incidents have higher case clearance rates than all other violent crimes included in the analyses. Beyond some influence of MRTCC information technologies, this improvement in clearance rates may be attributed to the following two elements: the relationship between the victim and offender and mandatory arrest policies. In all domestic violence incidents included within the analyses, a relationship existed between the victim and offender (i.e., family or intimate partner), facilitating the identification and apprehension of the perpetrator by law enforcement personnel. The prompt arrest of the offender may further encourage victims to cooperate with law enforcement and the prosecution, leading to a higher likelihood of successful case clearance or resolution. Moreover, improvement in clearance rates may be credited to the mandatory arrest policy implemented across Florida agencies. This policy requires officers to make an arrest when responding to a domestic violence call if they have probable cause to believe a crime has been committed, thus eliminating officer discretion.

Thirdly, despite the integration of MRTCC technologies with conventional evidentiary sources in processing other violent crimes, traditional means of evidence collection and crime scene analysis are found to be more prevalent in the investigation of sexual assault and domestic violence incidents. This discovery emphasizes the complexity of these crimes and the necessity for a multi-faceted investigative approach, where technological advancements supplement, rather than replace traditional investigative techniques. This finding underscores the importance of continually evaluating and improving investigative methods to ensure that the highest possible clearance rates for sexual assault and domestic violence cases are achieved.

The limitations of the study were its focus on only one city-level jurisdiction and a subset of criminal activity (i.e., violence). Accordingly, we do not know if these findings would hold up if replicated in other jurisdictions across the country, nor do we know how the use of these technologies for sexual assault by domestic violence investigations would compare to other non-violent crime types (i.e., property crimes). However, this does not diminish the significance of its findings. By elucidating the mechanisms by which RTCC platforms can successfully combat underreported criminal acts such as sexual assault and domestic violence, law enforcement agencies in other jurisdictions can potentially apply these approaches to bolster their efforts toward crime reduction and enhanced public safety.

Furthermore, in the context of case processing and outcomes, a significant limitation arose regarding the ability to determine the admissibility of evidence obtained by MRTCC detectives and external unit investigators. Specifically, the coding of variables was based solely on whether these personnel utilized sources of evidence or technologies, without considering whether such evidence ultimately proved to be valuable in the investigation process. For instance, although the dataset included CCTV footage, the actual evidentiary value of the footage was not taken into account. Additionally, the limited sample sizes prevented the independent analysis of domestic violence and sexual assault incidents.

## 5. Conclusions

The critical role of law enforcement in the development of a comprehensive strategy to combat sexual assault and domestic violence cannot be overstated. The enactment of the Violence Against Women Act in 1994 marked a significant milestone in the history of combating gender-based violence in the United States, with law enforcement at the forefront of its implementation [24]. This legislation, combined with advancements in technologies and techniques, has allowed police personnel to leverage resources to refine their investigative methods and identify perpetrators of sexual assault and domestic violence more effectively. However, despite these advancements, there remains a limited understanding of the impact that information technologies have on the processing and clearance of these incidents. The results of this study further highlight the distinctive features associated with this form of violent crime and underscore the necessity of continuously advancing the strategies employed to address these incidents. In short, while the greater use of information technologies by police may improve crime outcomes overall, they may not be the most beneficial way forward for incidents involving violence against women.

## Figures and Tables

**Table 1:** Comparative Overview of Measures Used in Nested Multivariate Logistic Regression Model and Module Network Analysis.

	Full Violent Crime Model (N = 1222)		Sexual Assaults (n = 42)		Domestic Violence (n = 84)	
Measures	RTCC-Assisted Cases n (%)	Control Cases n (%)	RTCC-Assisted Cases n (%)	Control Cases n (%)	RTCC-Assisted Cases n (%)	Control Cases n (%)
Cleared ^2^	237 (39%)	202 (33%) *	6 (29%)	4 (19%)	36 (86%)	33 (79%)
MRTCC technologies						
CCTV	525 (86%)	--	14 (67%)	--	35 (83%)	--
Social media	123 (20%)	--	9 (43%)	--	8 (19%)	--
Facial recognition	159 (26%)	--	9 (43%)	--	10 (24%)	--
ShotSpotter	113 (19%)	--	--	--	4 (10%)	--
License plate reader	75 (12%)	--	3 (14%)	--	--	--
Victim characteristics						
Victim age ^1^	35 (14.5) **	38 (16.2) **	27 (16.9)	23 (16.8)	32 (9.1) *	37 (13.0) *
Victim gender (female) ^2^	211 (35%)	245 (40%)	19 (91%)	19 (91%)	30 (71%)	30 (71%)
Victim race (Black) ^2^	317 (53%)	290 (47%)	8 (38%)	4 (20%)	30 (71%)	27 (64%)
Human evidence						
Victim interview ^2^	561 (92%)	571 (92%)	21 (100%)	21 (100%)	41 (98%)	42 (100%)
Witness interview ^2^	221 (36%) *	190 (31%) *	10 (48%)	7 (33%)	9 (21%)	12 (29%)
Lineup ^2^	141 (23%) **	102 (16%) **	7 (33%) *	16 (76%) *	1 (2%)	2 (5%)
Flyer ^2^	191 (31%) ***	54 (9%) ***	9 (43%)	1 (5%)	2 (5%)	1 (2%)
External CCTV ^2^	286 (47%) ***	71 (11%) ***	10 (48%) **	2 (10%) **	2 (5%)	--
Crime scene evidence						
Gun used ^1^	292 (48%) ***	185 (30%) ***	2 (10%)	--	7 (17%)	4 (10%)
Vehicle involved ^2^	353 (58%) ***	189 (30%) ***	10 (48%) **	1 (5%) **	24 (57%) ***	7 (17%) ***
Gunshot residue ^2^	72 (12%) ***	22 (4%) ***	--	31 (5%)	3 (7%)	--
Latent prints/DNA ^2^	147 (24%)	158 (25%)	11 (52%)	15 (71%)	2 (5%)	1 (2%)
CSI ^2^	327 (54%) ***	228 (37%)	12 (57%)	13 (62%)	7 (17%)	11 (26%)
NIBIN ^2^	171 (28%) ***	42 (7%) ***	--	--	5 (12%)	--
Warrant/manpower evidence						
Search warrant ^2^	47 (8%) ***	12 (2%) ***	2 (10%)	--	1 (2%)	--
Cell warrant ^2^	38 (6%) ***	9 (1%) ***	4 (19%)	--	1 (2%)	--
Number of officers ^1^	4.02 (1.87) **	3.74 (1.33) **	3.81(1.50)	4.24(1.04)	3.31(1.60)	3.21(0.871)

* *p* ≤ 0.05, ** *p* ≤ 0.01, *** *p* ≤ 0.001. ^1^ For these measures, independent samples *t*-tests were used to determine differences, equal variances not assumed. ^2^ Fisher’s Exact Test was used to determine differences for these dichotomous measures.

**Table 2:** Nested Multivariate Logistic Regression of Predictors of Case Clearance for Violent Crimes against Women.

Measures (N = 1222)	Model One B (Odds Ratio)	Model Two B (Odds Ratio)	Model Three B (Odds Ratio)	Model Four B (Odds Ratio)	Model Five B (Odds Ratio)	Model Six B (Odds Ratio)
VAW						
Sexual assault	−0.408 (0.665)	−0.415 (0.660)	−0.657 (0.518)	−1.370 (0.259) ***	−1.545 (0.213) ***	−1.624 (0.197) ***
Domestic violence	2.249 (9.482) ***	2.259 (9.575) ***	2.161 (8.681) ***	2.466 (11.774) ***	2.351 (10.492) ***	2.360 (10.590) ***
Technology						
MRTCC-assisted cases		0.302 (1.352) *	0.324 (1.382) *	0.326 (1.386) *	0.429 (1.536) **	0.395 (1.484) *
Victim characteristics						
Victim age			−0.002 (0.998)	0.003 (1.003)	0.000 (1.000)	0.000 (1.000)
Victim gender (female)			0.338 (1.402) *	0.270 (1.310)	0.155 (1.167)	0.157 (1.170)
Victim race (Black)			−0.125 (0.883)	−0.111 (0.895)	−0.032 (0.968)	−0.025 (0.976)
Human evidence						
Victim interview				0.645 (1.905) *	0.471 (1.602)	0.517 (1.677)
Witness interview				0.496 (1.641) ***	0.490 (1.632) ***	0.469 (1.599) **
Lineup				1.717 (5.570) ***	1.737 (5.679) ***	1.758 (5.802) ***
Flyer				0.616 (1.852) **	0.684 (1.982) ***	0.616 (1.852) **
External CCTV				−0.807 (0.499) ***	−0.756 (0.469) ***	−0.831 (0.436) ***
Crime scene evidence						
Gun used					−0.675 (0.509) ***	−0.668 (0.513) ***
Vehicle used					−0.192 (0.826)	−0.265 (0.767)
Gunshot residue (GSR)					0.189 (1.208)	0.148 (1.160)
Latent prints/DNA					−0.084 (0.919)	−0.184 (0.832)
CSI					0.056 (1.058)	0.040 (1.041)
Ballistics tracing (NIBIN)					0.009 (1.009)	−0.007 (0.993)
Warrant evidence and manpower						1.083 (2.953) **
Search warrant						0.670 (1.954)
Cell warrant						0.015 (1.015)
Number of officers						1.083 (2.953) **
Chi-square model	85.349 ***	89.376 ***	97.075 ***	251.539 ***	274.656 ***	295.076
−2 Log likelihood	1513.054	1507.129	1499.430	1344.966	1321.849	1301.429
Nagelkerke pseudo R-square	0.090	0.097	0.105	0.255	0.276	0.294
Constant	−0.585(0.557) ***					

* *p* ≤ 0.05, ** *p* ≤ 0.01, *** *p* ≤ 0.001.

**Table 3:** Post Hoc Comparative Overview of Traditional Extralegal Measures Used in Analysis (VAW only).

	Sexual Assaults n = 42		Domestic Violence n = 84	
Measures (N = 126)	MRTCC-Assisted Cases n (%)	Control Cases n (%)	MRTCC-Assisted Cases n (%)	Control Cases n (%)
Victim characteristics				
Victim age ^1^	27 (16.9)	23 (16.8)	32 (9.1) *	37 (13.0) *
Victim gender (female) ^2^	19 (91%)	19 (91%)	30 (71%)	30 (71%)
Victim race (Black) ^2^	8 (38%)	4 (20%)	30 (71%)	27 (64%)
Victim offender relationship				
Family ^2^	2 (10%)	6 (29%)	3 (7%)	8 (19%)
Intimate partner ^2^	3 (14%)	5 (24%)	39 (93%)	33 (79%)
Stranger ^2^	16 (76%)	9 (43%)	--	--
Location				
Public space ^2^	14 (67%)	9 (43%)	23 (55%)	16 (38%)
Residence ^2^	6 (29%)	10 (48%)	18 (43%)	27 (64%)
Crime severity				
Weapon used ^2^	--	--	20 (48%)	15 (36%)
Visible injury ^2^	1 (5%)	1 (5%)	20 (48%)	18 (43%)
Under influence ^2^	4 (19%)	7 (33%)	4 (10%)	7 (17%)
Prior record ^2^	1 (5%)	--	11 (26%)	17 (41%)

* *p* ≤ 0.05; ^1^ For these measures, independent samples *t*-tests were used to determine differences; equal variances not assumed. ^2^ Fisher’s Exact Test was used to determine differences for these dichotomous measures.

**Table 4:** Post Hoc Non-Nested Multivariate Logistic Regression of Traditional Predictors of Case Clearance.

Measures (N = 126)	Model One B (Odds Ratio)	Model Two B (Odds Ratio)	Model Three B (Odds Ratio)	Model Four B (Odds Ratio)	Model Five B (Odds Ratio)	Model Six B (Odds Ratio)
VAW						
Sexual assault	−2.689 (0.068) ***	−2.727 (0.065) ***	−2.960 (0.052) ***	−2.902 (0.055) ***	−2.668 (0.069) ***	−2.798 (0.061) ***
Technology						
MRTCC-assisted cases		0.507 (1.660)	0.554 (1.740)	0.593 (1.809)	0.549 (1.732)	0.454 (1.575)
Victim characteristics						
Victim age			0.010 (1.010)			
Victim gender (female)			0.438 (1.549)			
Victim race (Black)			−0.475 (0.622)			
Relationship						
Family				0.399 (1.490)		
Intimate partner				−0.277 (0.758)		
Stranger				−0.102 (0.903)		
Location						
Public space					1.121 (3.067)	
Residence					1.217 (3.376)	
Crime severity						
Weapon used						0.698 (2.010)
Visible injury						0.279(1.322)
Under influence						0.536 (1.709)
Prior record						−0.861 (0.423)
Chi-square model	41.522 ***	42.783 ***	43.191 ***	43.680 ***	43.630 ***	47.988 ***
−2 Log likelihood	124.934	123.674	121.279	122.777	122.827	118.468
Pseudo R-square	0.383	0.393	0.399	0.400	0.399	0.432
Constant	0.519 (1.681) **	0.519 (1.681) **	0.541 (1.717) **	0.519 (1.681) **	0.519 (1.681) **	0.519 (1.681) **

** *p* ≤ 0.01, *** *p* ≤ 0.001.

## Data Availability

The data presented in this study are available on request from the corresponding author. The data are not publicly available due to data agreement with Miami Police Department.
